# Complement factor B in high glucose–induced podocyte injury and diabetic kidney disease

**DOI:** 10.1172/jci.insight.147716

**Published:** 2021-10-08

**Authors:** Qingmiao Lu, Qing Hou, Kai Cao, Xiaoli Sun, Yan Liang, Mengru Gu, Xian Xue, Allan Zijian Zhao, Chunsun Dai

**Affiliations:** 1 Center for Kidney Disease and; 2 Department of Clinical Genetics, Second Affiliated Hospital, Nanjing Medical University, Nanjing, China.; 3 Institute of Biomedical and Pharmaceutical Sciences, Guangdong University of Technology, Guangzhou, China.

**Keywords:** Inflammation, Nephrology, Chronic kidney disease, Complement

## Abstract

The role and mechanisms for upregulating complement factor B (CFB) expression in podocyte dysfunction in diabetic kidney disease (DKD) are not fully understood. Here, analyzing Gene Expression Omnibus GSE30528 data, we identified genes enriched in mTORC1 signaling, CFB, and complement alternative pathways in podocytes from patients with DKD. In mouse models, podocyte mTOR complex 1 (mTORC1) signaling activation was induced, while blockade of mTORC1 signaling reduced CFB upregulation, alternative complement pathway activation, and podocyte injury in the glomeruli. Knocking down CFB remarkably alleviated alternative complement pathway activation and DKD in diabetic mice. In cultured podocytes, high glucose treatment activated mTORC1 signaling, stimulated STAT1 phosphorylation, and upregulated CFB expression, while blockade of mTORC1 or STAT1 signaling abolished high glucose–upregulated CFB expression. Additionally, high glucose levels downregulated protein phosphatase 2Ac**α** (PP2Ac**α**) expression, while PP2Ac**α** deficiency enhanced high glucose–induced mTORC1/STAT1 activation, CFB induction, and podocyte injury. Taken together, these findings uncover a mechanism by which CFB mediates podocyte injury in DKD.

## Graphical abstract

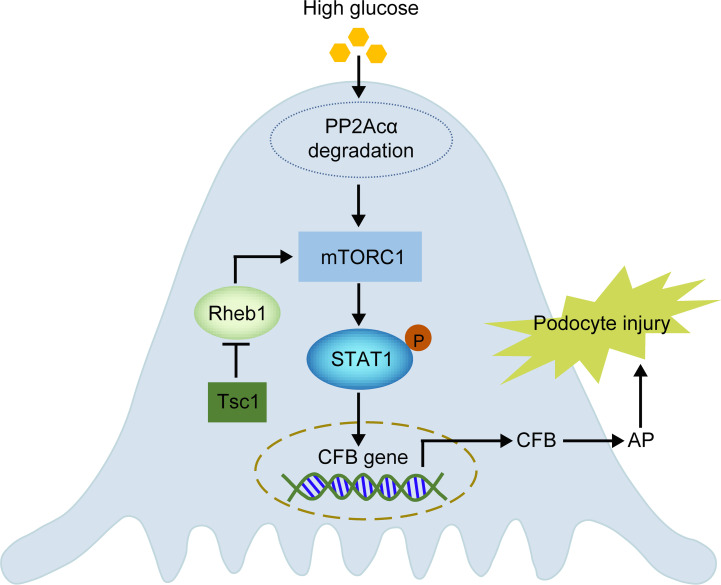

## Introduction

Millions of people worldwide have diabetes mellitus, and approximately 20%–40% of them develop diabetic kidney disease (DKD) (1–4). DKD has become a major cause of chronic kidney disease and end-stage renal disease (5). Deciphering the pathogenesis of DKD is an unmet medical need that is crucial for developing new therapies for DKD prevention and treatment.

Studies of both clinical and experimental DKD implicate podocyte injury and dysfunction as resulting in albuminuria and glomerulosclerosis (6–8). Immune-mediated inflammation, especially activation of the complement system (a major player in innate and adaptive immunity), has been increasingly recognized as a pathogenic factor for podocyte injury in DKD (9–12). How the complement system is aberrantly activated in DKD remains poorly understood. While the complement system is typically activated through the classic pathway and the lectin pathway, the CFB-regulated alternative pathway plays a significant role in the pathogenesis of kidney diseases, such as lupus nephritis (13, 14), atypical hemolytic uremic syndrome (15, 16), complement 3 (C3) glomerulopathies (GNs) (17), anti-neutrophil cytoplasmic antibody–associated (ANCA-associated) GN (18, 19), autosomal dominant polycystic kidney disease (ADPKD) (20), IgA nephropathy (21), and immune rejection after renal transplantation (22). Activation of these pathways leads to the production of C3 convertase and, eventually, the formation of a membrane attack complex, which destroys target cells through lytic and sublytic effects (23). Unlike the classic and lectin pathways, the complement alternative pathway is activated by the slow, spontaneous “tickover” mechanism of spontaneous hydrolysis of the thioester bond in C3 (24, 25). Complement factor B (CFB) is the key regulator of this process. The cleavage product Bb combines with C3b to form C3 invertase for cleaving C3, which forms a positive feedback loop to continuously activate the alternative complement pathway (24, 26). Therefore, more attention has been given to determining the role of complement activation in DKD (27, 28). However, the regulatory mechanisms of activating the alternative complement pathway in podocytes in DKD remain to be determined.

The mammalian target of rapamycin (mTOR) is an evolutionarily conserved serine/threonine protein kinase belonging to the PI3K-related protein kinase family. It participates in forming 2 distinct complexes, mTOR complex 1 (mTORC1) and mTOR complex 2. mTORC1 is a rapamycin-sensitive protein kinase complex and regulates a wide array of cellular processes, including cell growth, proliferation, and autophagy in response to nutrients such as glucose, amino acids, and growth factors (29–33). Previous findings suggest that mTORC1 plays a role in podocyte dysfunction and DKD, and we found that mTORC1 elevation in Tsc1-deleted macrophages significantly upregulated CFB expression, suggesting that mTORC1 may stimulate complement alternative pathway activation to promote podocyte injury and DKD.

In this study, we demonstrated that CFB upregulation enhanced alternative complement pathway activation in the glomeruli during the development of DKD in both patients and mouse models, which was correlated with mTORC1 activation and diabetic glomerulosclerosis. We found that CFB expression is upregulated by genetic activation of mTORC1 in podocytes, which promoted alternative complement pathway activation and podocyte injury. Moreover, we demonstrated that high glucose–downregulated protein phosphatase 2Acα (PP2Acα) enhanced mTORC1 activation and podocyte injury. These findings support the notion that high glucose could upregulate CFB expression and enhance activation of the alternative complement pathway through mTORC1 activation, which in turn promotes podocyte injury and DKD.

## Results

### Induction of CFB and activation of the alternative complement pathway in podocytes from DKD patients and mice.

**Figure 1:**
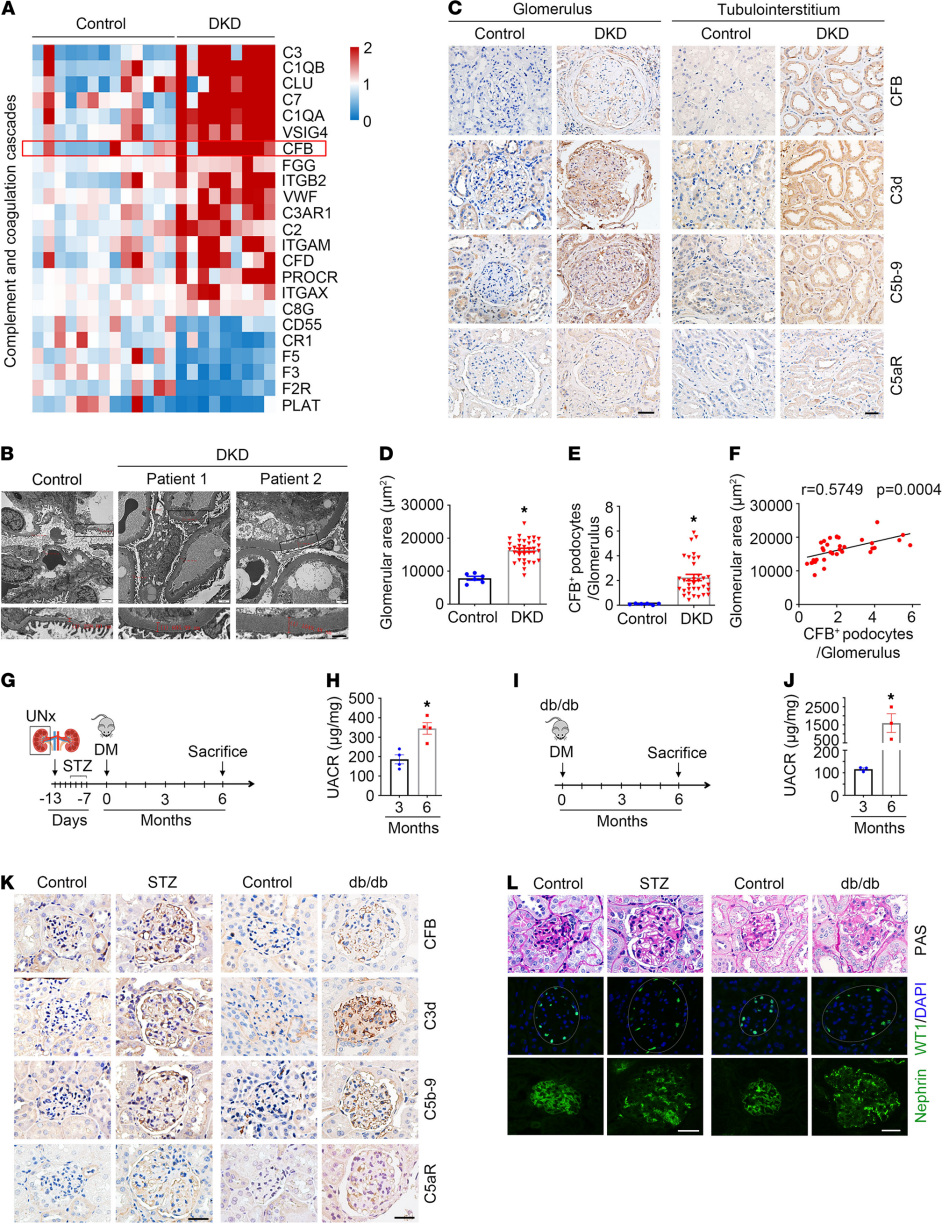
Activation of alternative complement pathway in glomeruli from DKD patients and mice. (**A**) Heatmap of the differentially expressed genes enriched in alternative complement pathway in glomeruli from human kidneys with DKD. (**B**) Representative transmission electron microscopy (TEM) pictures showing podocyte foot process effacement and glomerular basement membrane (GBM) thickness, represented by red text, in patients with DKD. Scale bars: 2 μm (above), 1 μm (below). (**C**) Representative immunohistochemical staining images showing the induction of CFB, C3d, C5b-9, and C5aR in glomerulus and tubulointerstitium from DKD renal biopsies. Scale bar: 20 μm. (**D**) Quantitative analyses of glomerular area in DKD renal biopsies. *P < 0.05 (control, *n* = 6; DKD, *n* = 34). (**E**) Quantitative analyses of CFB-staining-positive podocytes per glomerulus in renal biopsies. *P < 0.05 (control, *n* = 6; DKD, *n* = 34). (**F**) Linear correlation and regression analyses showing a significant positive correlation between glomerular area and CFB expression in DKD renal biopsies (*n* = 34). (**G**) The strategy for establishing a mouse model of DKD. (**H**) UACR 3 and 6 months after DM. *P < 0.05, *n* = 4. (**I** and **J**) UACR from db/db mice 3 and 6 months after DM. *P < 0.05, *n* = 3. (**K**) Representative immunohistochemical staining images showing the induction of CFB, C3d, C5b-9, and C5aR in glomeruli of STZ-treated mice and db/db mice. Scale bar: 20 μm. (**L**) Representative periodic acid–Schiff (PAS) staining and representative immunofluorescence staining revealing a decreased WT1-positive podocyte number and reduced nephrin abundance in STZ-treated mice and db/db mice. Scale bar: 20 μm. Data are expressed as the mean ± SEM. Comparison between the groups was performed using the 2-tailed Student’s *t* test (unpaired *t* test). Pearson’s correlation was used to determine relationships between variables. UACR, urinary albumin-to-creatinine ratio.

To explore the role of the alternative complement activation pathway in DKD, we analyzed Gene Expression Omnibus GSE30528 data obtained from glomeruli of 13 human DKD kidneys and 9 controls uploaded (34). The results revealed genes enriched in complement system activation via Kyoto Encyclopedia of Genes and Genomes (KEGG) and Gene Ontology (GO) analysis, such as C3, C1QB, CLU, C7, C1QA, VSIG4, CFB, FGG, ITGB2, VWF, C3AR1, C2, ITGAM, CFD, PROCR, ITGAX, C8G, CD55, CR1, F5, F3, F2R, and PLAT. Among them, CFB, a key positive regulator of the alternative complement activation pathway, was markedly upregulated (Figure 1A).

To further examine activation of the alternative complement pathway in podocytes of DKD kidneys, we stained renal biopsies from patients with DKD and adjacent healthy kidney tissues of kidney tumor patients with antibodies against CFB, C3d, C5b-9, and C5aR. General patient information is shown in Supplemental Table 1; supplemental material available online with this article; https://doi.org/10.1172/jci.insight.147716DS1 Compared with those in the control kidneys, DKD biopsies exhibited obvious podocyte foot process effacement, thickening glomerular basement membranes (control: 228.80 nm, DKD patient 1: 605.99 nm, DKD patient 2: 1049.48 nm) (Figure 1B), and significantly increased glomerular area (Figure 1, C and D). Immunohistochemical staining confirmed the remarkable elevation of CFB, C3d, C5b-9, and C5aR abundance in DKD glomeruli and tubules compared with control kidneys (Figure 1, C and E). Notably, a significantly positive correlation between CFB induction in podocytes and glomerular enlargement was observed (Figure 1F).

**Figure 2:**
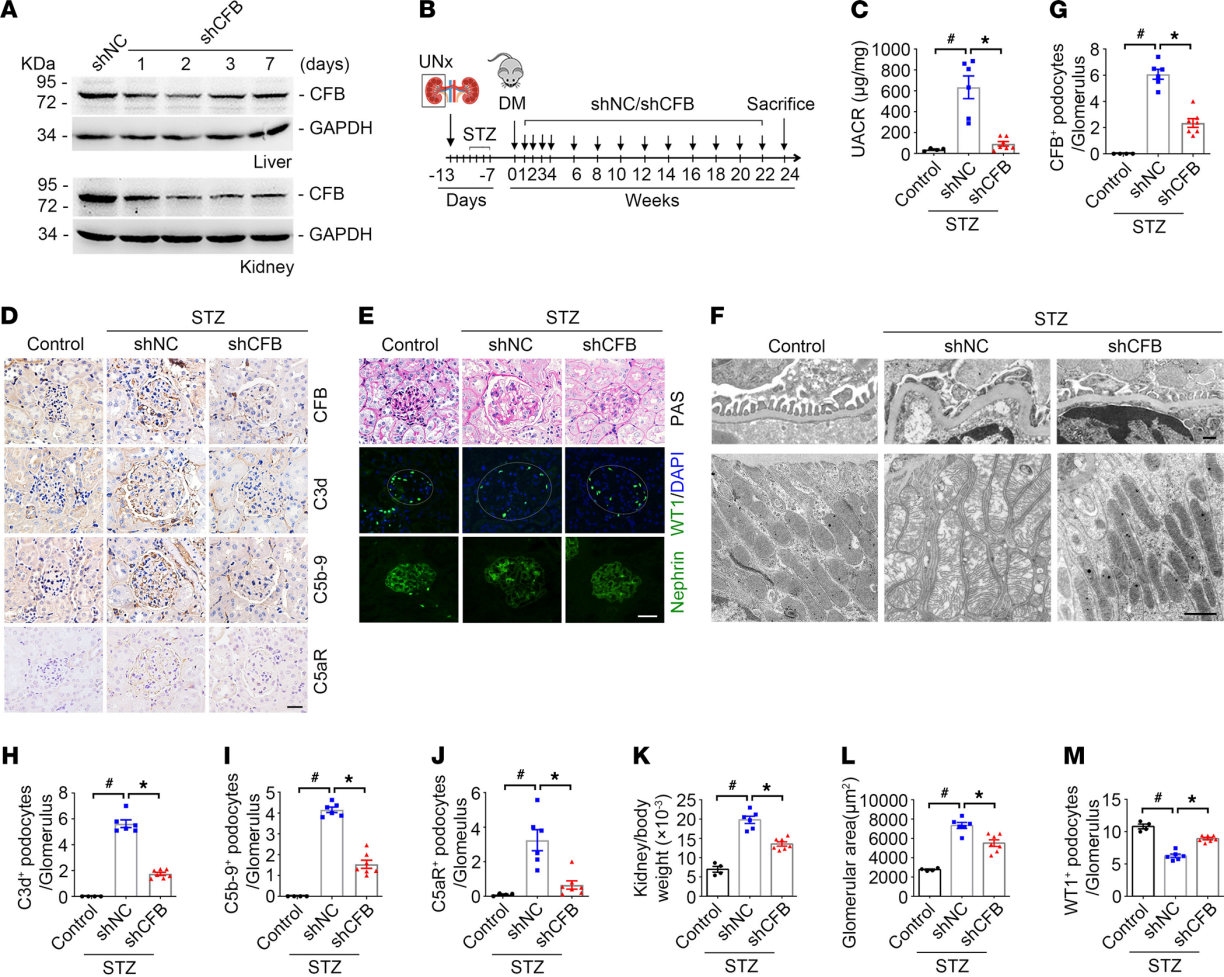
CFB knockdown attenuates podocyte injury and DKD in STZ-induced diabetic mice. (**A**) Western blot assay showing CFB expression in mouse livers and kidneys after CFB shRNA (shCFB) injection. shNC, scrambled shRNA. (**B**) The strategy for establishing a mouse model of DKD and the injection of shCFB. (**C**) UACR among different groups. ^#^*P* < 0.05, *P < 0.05, *n* = 4–7. (**D**) Representative immunohistochemical staining images showing the abundance of CFB, C3d, C5b-9, and C5aR in glomerulus among different groups. Scale bar: 20 μm. (**E**) Representative PAS staining and representative immunofluorescence staining images for diabetic kidney injury, WT1, and nephrin among different groups. Scale bar: 20 μm. (**F**) Representative TEM images. Scale bar: 400 nm (above). Scale bar: 1 μm (below). (**G**–**J**) Quantitative analyses of CFB, C3d, C5b-9, and C5aR-staining per glomerulus among different groups. ^#^*P* < 0.05, *P < 0.05, *n* = 4–7. (**K**) The graphs showing the kidney/body weight ratio among different groups. ^#^*P* < 0.05, *P < 0.05, *n* = 4–7. (**L** and **M**) Quantitative analyses of glomerular area (**L**) and WT1-positive podocytes per glomerulus (**M**) among different groups. ^#^*P* < 0.05, *P < 0.05, *n* = 4–7. Data are expressed as the mean ± SEM. Comparison between the groups was performed using 1-way ANOVA followed by the Tukey test.

Then, in streptozotocin-induced (STZ-induced) type 1 diabetic mice and db/db mice, which developed albuminuria 3 months and 6 months after diabetes mellitus (DM) (Figure 1, G–J), obvious induction of CFB, C3d, C5b-9, and C5aR was observed in the glomeruli 6 months after DM by immunohistochemical staining (Figure 1K; Figure 2, G–J; and Supplemental Figure 2, F–I). In line with alternative complement pathway activation, an enlarged glomerular area, decreased Wilms tumor 1–positive (WT1-positive) podocyte number, and nephrin redistribution were observed in DKD kidneys (Figure 1L). Collectively, these data reveal that induction of CFB and excessive activation of the alternative complement pathway in glomeruli are correlated with DKD.

### Knocking down CFB expression attenuates podocyte injury and DKD in mice.

To explore the role of CFB induction in podocyte injury and DKD, we knocked down CFB expression using CFB shRNA plasmid injection in mice. The results of the Western blot assay and immunohistochemical staining showed that CFB protein abundance in the liver and kidney was remarkably decreased 2 days after CFB shRNA injection and remained at lower levels for at least 7 days (Figure 2A and Supplemental Figure 1, A and B).

In control mice injected with CFB shRNA, no significant differences in body or organ weight; random blood glucose; serum lipid parameters of triglycerides, total cholesterol, and LDL-cholesterol (LDL-C); or kidney function (UACR and serum creatinine, SCr) or structure were observed compared to mice injected with scrambled shRNA (Supplemental Figure 1, C, D, and F–J). However, we observed decreased intraperitoneal glucose tolerance and increased HDL-C levels in mice with CFB knockdown compared with those injected with scrambled shRNA (Supplemental Figure 1, E and H). This finding needs further study to confirm whether CFB knockdown has an effect on energy metabolism in mice.

In STZ-induced DM mice with CFB knockdown (Figure 2B), there were no significant differences in body weight, blood glucose, or serum lipid parameters compared to STZ-induced DM mice without CFB knockdown (Supplemental Figure 1, K–M). Additionally, reduced proteinuria and SCr levels were observed in STZ-induced DM mice with CFB knockdown than in those injected with scrambled shRNA (Figure 2C and Supplemental Figure 1N). The induction of CFB, C3d, C5b-9, and C5aR in the glomeruli of STZ-induced diabetic kidneys was significantly reduced in mice injected with CFB shRNA (Figure 2, D and G–J). Additionally, the increased kidney weight, enlarged glomerular area, decreased WT1-positive podocytes, and nephrin redistribution were significantly alleviated in mice by CFB knockdown (Figure 2, E and K–M). TEM examination revealed that the effacement of podocyte foot processes and severe thickening and irregular shape of the GBM were largely ameliorated by CFB knockdown (average thickness of GBM: control 166.16 nm, STZ+shNC 577.26 nm, STZ+shCFB 196.98 nm), and the mitochondrial damage in tubules from STZ-induced DKD mice was also obviously ameliorated in response to CFB knockdown (percentage of damaged mitochondria: STZ+shNC 63%, STZ+shCFB 22%) (Figure 2F). The data showed that knocking down CFB expression remarkably inhibited alternative complement pathway activation and attenuated DKD in STZ-induced diabetic mice.

In db/db mice, similar to results in STZ-injected mice, knocking down CFB expression had no effect on body weight, blood glucose, or serum lipid parameters (Supplemental Figure 1, O–Q, and Supplemental Figure 2A) but significantly diminished albuminuria and SCr levels (Supplemental Figure 2B and Supplemental Figure 1R); reduced the induction of CFB, C3d, C5b-9, and C5aR in glomeruli (Supplemental Figure 2, C and F–I); decreased the enlarged glomerular area; restored WT1-positive podocyte number and membrane localization of nephrin (Supplemental Figure 2, D and J–L); and ameliorated the effacement of podocyte foot processes and severe thickening and irregular shape of the GBM (average thickness of GBM: control 167.28 nm, db/db+shNC 462.48 nm, STZ+shCFB 209.87 nm), as well as the mitochondrial damage in tubules (the percentage of damaged mitochondria: db/db+shNC 64%, db/db+shCFB 40%) (Supplemental Figure 2E). Therefore, these data suggest that knocking down CFB expression remarkably attenuates DKD in type 2 DM mice.

### mTORC1 mediates high glucose–upregulated CFB expression and alternative complement pathway activation in podocytes.

**Figure 3:**
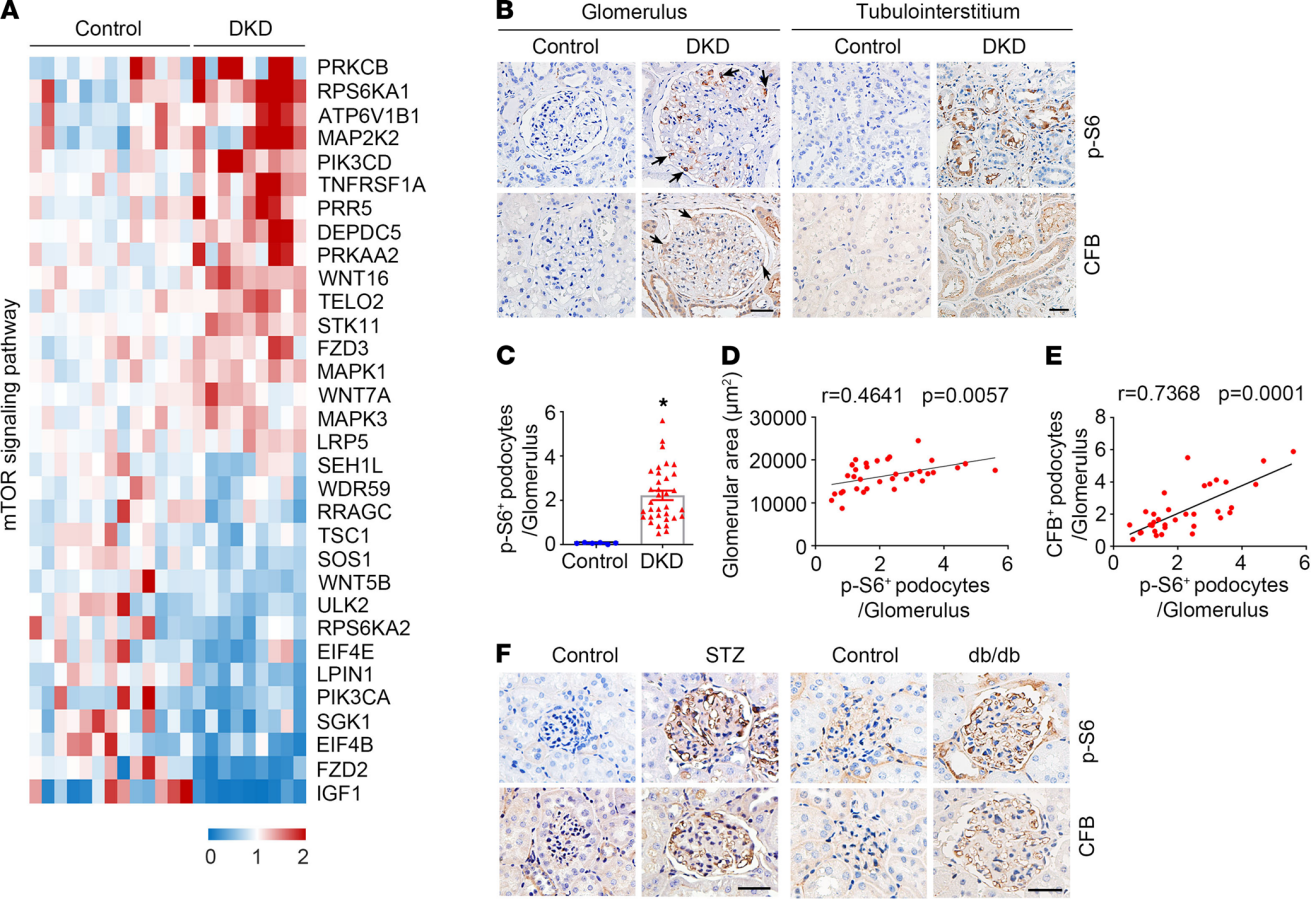
mTORC1 activation is associated with CFB upregulation in podocytes from DKD patients and mice. (**A**) Heatmap of the differentially expressed genes enriched in mTORC1 signal pathway in glomeruli from patients with DKD. (**B**) Representative immunohistochemical staining images showing the induction of p-S6 and CFB in glomerulus and tubulointerstitium from DKD renal biopsies. Scale bar: 20 μm. (**C**) Quantitative analyses of p-S6-staining-positive podocytes per glomerulus in DKD renal biopsies. *P < 0.05 (control, *n* = 6; DKD, *n* = 34). (**D**) Linear correlation and regression analyses showing a significant positive correlation between glomerular area and p-S6 expression in DKD renal biopsies (*n* = 34). (**E**) Linear correlation and regression analyses showing a significant positive correlation between CFB expression and p-S6 expression in DKD renal biopsies (*n* = 34). (**F**) Representative immunohistochemical staining images showing the induction of p-S6 and CFB in glomerulus in STZ-treated mice and db/db mice. Scale bar: 20 μm. Data are expressed as the mean ± SEM. Comparison between the groups was performed using the 2-tailed Student’s *t* test (unpaired *t* test). Pearson’s correlation was used to determine relationships between variables.

The above data show that CFB expression was upregulated in podocytes in the setting of DKD; however, how CFB upregulation is modulated under high-glucose conditions remains unclear. Previous studies revealed that podocyte-specific mTORC1 activation promotes podocyte injury and DKD (35, 36). By analyzing the GSE30528 data from glomeruli of patients with DKD, differentially expressed genes enriched in the mTORC1 signaling pathway, including PRKCB, RPS6KA1, ATP6V1B1, MAP2K2, PIK3CD, TNFRSF1A, PRR5, DEPDC5, PRKAA2, WNT16, TELO2, STK11, FZD3, MAPK1, WNT7A, MAPK3, LRP5, SEH1L, WDR59, RRAGC, TSC1, SOS1, WNT5B, ULK2, RPS6KA2, EIF4E, LPIN1, PIK3CA, SGK1, EIF4B, FZD2, and IGF1, were identified (Figure 3A). Immunohistochemistry staining results showed the induction of p-S6 in glomeruli of DKD biopsies, which was positively correlated with glomerular enlargement (Figure 3, B–D). Importantly, a significant positive correlation was observed between p-S6 abundance and CFB induction in DKD renal biopsies (Figure 3E). Similarly, mTORC1 signaling was activated in podocytes from STZ-injected mice and db/db mice (Figure 3F). Therefore, these data suggest that mTORC1 may upregulate CFB expression in podocytes in both patients with DKD and animal models.

**Figure 4:**
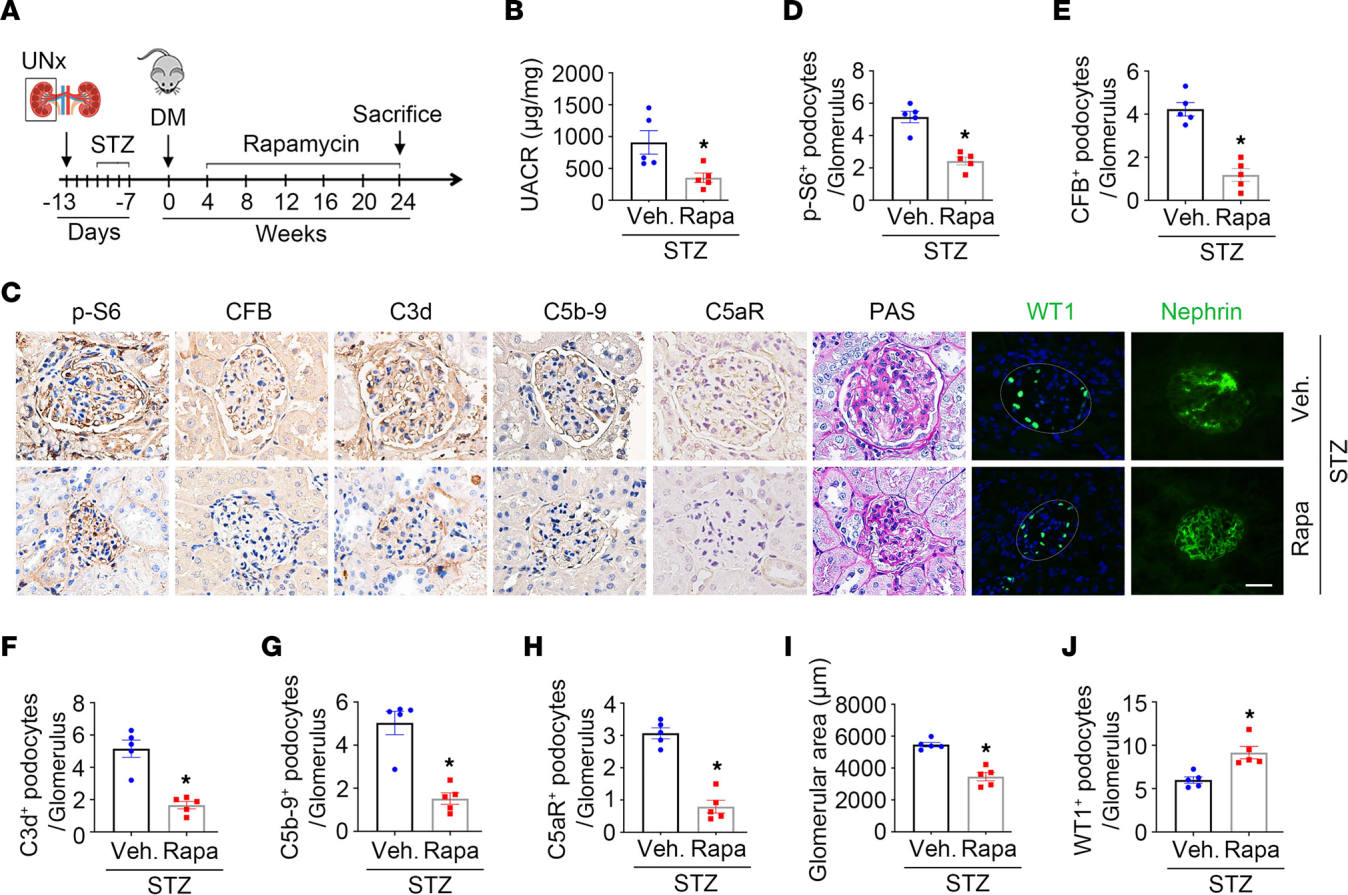
Blocking mTORC1 with rapamycin attenuates alternative complement pathway activation and podocyte injury in STZ-induced diabetic mice. (**A**) The strategy for STZ injection and rapamycin administration. (**B**) UACR among different groups. *n* = 5. (**C**) Representative immunohistochemical staining images for p-S6, CFB, C3d, C5b-9, and C5aR in glomerulus among different groups. Representative PAS staining and immunofluorescence staining for diabetic kidney injury, WT1, and nephrin among different groups. Scale bar: 20 μm. (**D**–**J**) Quantitative analyses of p-S6, CFB, C3d, C5b-9, C5aR, and WT1-positive podocytes per glomerulus and glomerular area among different groups. *P < 0.05, *n* = 4–7. Data are expressed as the mean ± SEM. Comparison between the groups was performed using the 2-tailed Student’s *t* test (unpaired *t* test).

To further explore the role of mTORC1 activation in stimulating CFB upregulation and complement alternative pathway activation in podocytes, we treated STZ-injected mice with rapamycin to block the mTORC1 signaling pathway (Figure 4A). Consistent with previous reports, rapamycin markedly mitigated albuminuria (Figure 4B), glomerular hypertrophy, podocyte loss, and nephrin redistribution in STZ-induced DKD mice (Figure 4, C, I, and J). Notably, the increase in p-S6, CFB, C3d, C5b-9, and C5aR abundance in podocytes from mice with STZ-induced DKD was significantly reduced after rapamycin administration (Figure 4, C–H).

Rheb1 is a crucial activator of mTORC1 signaling (37). Tsc1, a stabilizer of Tsc2 that functions as a GTPase-activating protein for Rheb1, inhibits Rheb1 and mTORC1 signaling activation (38). To further decipher the role of mTORC1 signaling in upregulating CFB expression, we generated mouse models with podocyte-specific Tsc1 knockout (Podo-Tsc1^–/–^), podocyte-specific Rheb1 knockout (Podo-Rheb1^–/–^), and both Tsc1 and Rheb1 knockout (Supplemental Figure 3A). The albuminuria that developed in Podo-Tsc1^–/–^ mice was almost completely abolished in Podo-Tsc1^–/–^ and Rheb1^–/–^ mice and was not observed in Podo-Rheb1^–/–^ mice (Supplemental Figure 3B). Moreover, the induction of p-S6, CFB, C3d, C5b-9, and C5aR in Podo-Tsc1^–/–^ kidneys was significantly decreased in Podo-Tsc1^–/–^ and Rheb1^–/–^ as well as Podo-Rheb1^–/–^ kidneys (Supplemental Figure 3, C–I). Therefore, these data demonstrate that activated mTORC1 may upregulate CFB expression to promote alternative complement pathway activation in podocytes.

### STAT1 mediates mTORC1-upregulated CFB expression in podocytes.

**Figure 5:**
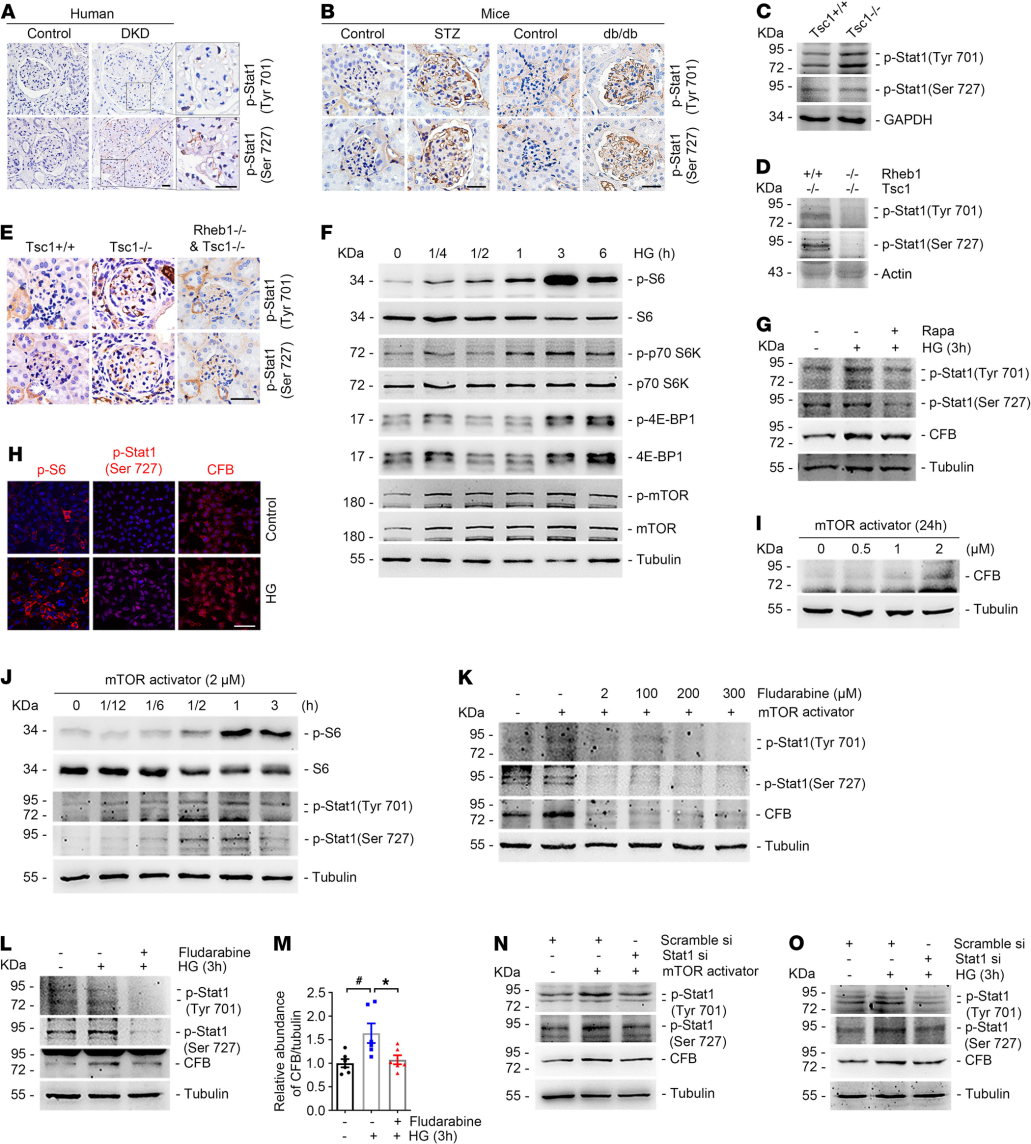
STAT1 mediates mTORC1-upregulated CFB expression in podocytes. (**A** and **B**) Representative immunohistochemical staining images showing the induction of p-STAT1 (Tyr701) and p-STAT1 (Ser727) in glomerulus from DKD human renal biopsies and mouse models. Scale bar: 20 μm. (**C** and **D**) Western blot assay showing the abundance of p-STAT1 (Tyr701) and p-STAT1 (Ser727) in mouse kidneys. (**E**) Representative immunohistochemical staining images showing the induction of p-STAT1 (Tyr701) and p-STAT1 (Ser727) in glomeruli among groups. Scale bar: 20 μm. (**F**) Western blot assay showing the abundance of p-S6, p-p70 S6K, p-4E-BP1, and p-mTOR in high glucose–cultured podocytes. (**G**) Western blot assay showing the abundance of p-STAT1 (Tyr701), p-STAT1 (Ser727), and CFB in high glucose–cultured podocytes treated with rapamycin (5 nM). (**H**) Representative immunofluorescence staining showing the abundance of p-S6, p-STAT1 (Ser727), and CFB in high glucose–cultured podocytes. Scale bar: 20 μm. (**I** and **J**) Western blot assay showing the abundance of CFB (**I**), p-S6, p-STAT1 (Tyr701), and p-STAT1 (Ser727) (**J**) in cultured podocytes treated with mTOR activator. (**K**) Western blot assay showing the abundance of p-STAT1 (Tyr701), p-STAT1 (Ser727), and CFB in cultured podocytes treated with fludarabine and mTOR activator (2 μM). (**L**) Western blot assay showing the abundance of p-STAT1 (Tyr701), p-STAT1 (Ser727), and CFB in high glucose–cultured podocytes treated with fludarabine (0.4 μM). (**M**) Quantitative analyses showing the abundance of CFB in high glucose–cultured podocytes treated with fludarabine (0.4 μM). ^#^*P* < 0.05, *P < 0.05, *n* = 3. (**N**) Western blot assay showing the abundance of p-STAT1 (Tyr701), p-STAT1 (Ser727), and CFB in cultured podocytes treated with STAT1 siRNA and mTOR activator (2 μM). (**O**) Western blot assay showing the abundance of p-STAT1 (Tyr701), p-STAT1 (Ser727), and CFB in high glucose–cultured podocytes treated with STAT1 siRNA. Data are expressed as the mean ± SEM. Comparison between the groups was performed using 1-way ANOVA followed by the Tukey test.

It has been reported that STAT1 phosphorylation on tyrosine 701 and serine 727 is required for its dimerization and full transcriptional activity, respectively (39, 40). Wu et al. revealed that STAT1 may upregulate CFB expression in renal epithelial cells (41). To explore whether mTORC1 regulates CFB expression by activating STAT1 signaling in podocytes, we first examined the phosphorylation status of STAT1 in kidneys from DKD patients and mice. Markedly increased phosphorylated STAT1 (p-STAT1) on serine 727, but not tyrosine 701, was detected in podocytes from patients with DKD (Figure 5A), while in DKD mice, the abundance of p-STAT1 on serine 727 and tyrosine 701 was both largely increased and accompanied by mTORC1 signaling activation in podocytes (Figure 5B). In the kidneys of mice with Tsc1 deletion in podocytes with enhanced mTORC1 activity, the STAT1 phosphorylation on serine 727 and tyrosine 701 was markedly increased, which was diminished after Rheb1 gene deletion (Figure 5, C–E).

In podocytes cultured with high glucose, the abundance of p-S6, p-p70 S6K, p-4E-BP1, and p-mTOR was increased in a time-dependent manner (Figure 5F). Consistently, STAT1 phosphorylation and CFB expression were remarkably increased in response to high glucose administration and were reduced after rapamycin treatment (Figure 5, G and H). Similar to high glucose treatment, in podocytes treated with mTOR activator, the abundance of p-S6, p-STAT1, and CFB was markedly increased in a time-dependent manner (Figure 5, I and J). Next, fludarabine, a STAT1 inhibitor, or STAT1 siRNA largely reduced the induction of p-STAT1 and CFB by mTOR activator or high glucose treatment (Figure 5, K–O). Collectively, these data suggest that STAT1 mediates mTORC1-induced CFB expression in podocytes under high glucose treatment.

### Rheb1 does not contribute to high glucose–induced mTORC1 activation in podocytes.

**Figure 6:**
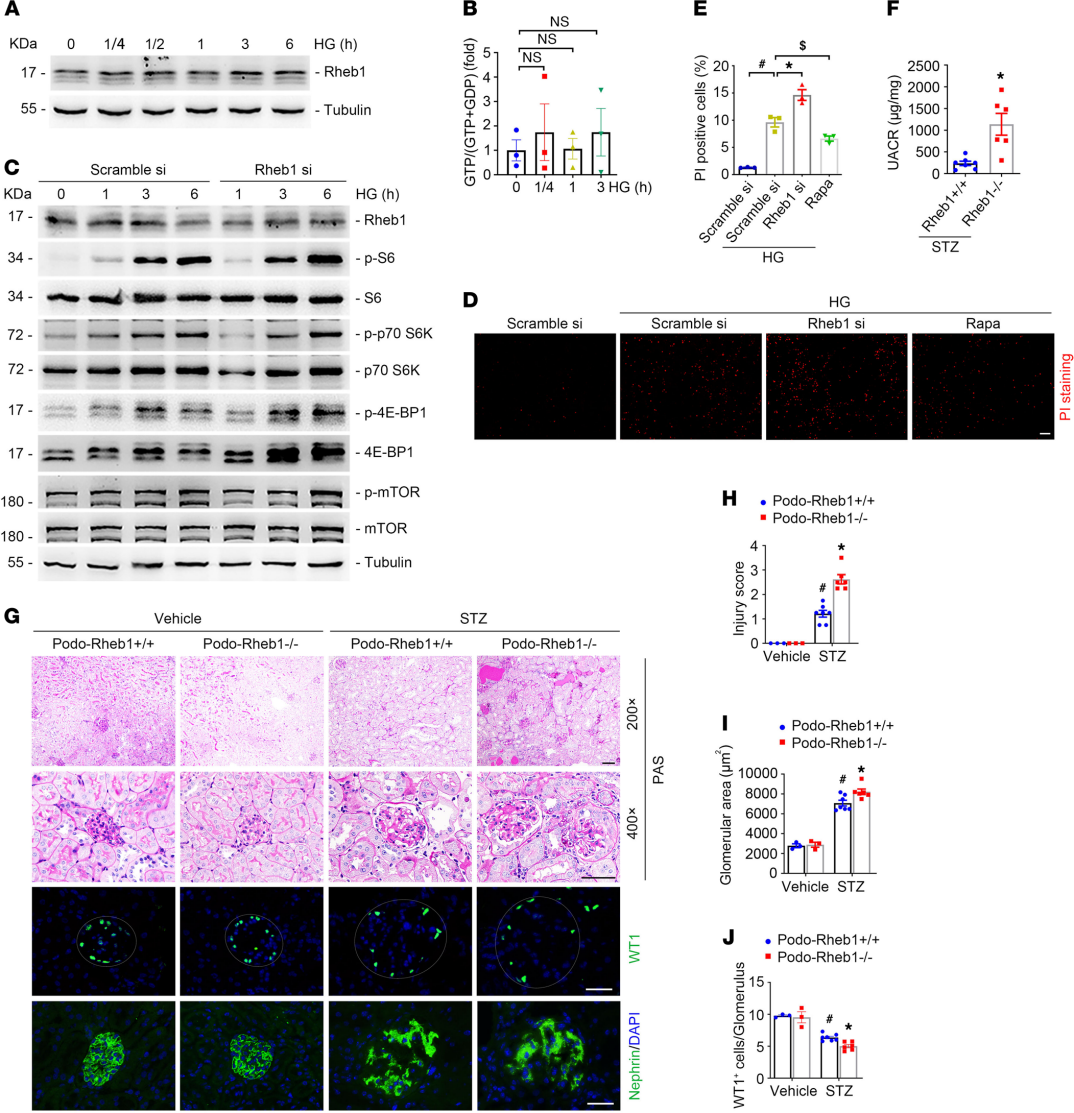
Rheb1 is dispensable for high glucose–induced mTORC1 activation, and ablation of Rheb1 aggravates podocyte injury in streptozotocin-induced diabetic mice. (**A**) Western blot assay showing the abundance of Rheb1 in cultured podocytes after high glucose treatment at different times. (**B**) GTP loading assay showing the induction of GTP-Rheb1 after high glucose treatment. (**C**) Western blot assay showing the abundance of p-S6, p-p70 S6K, p-4E-BP1, and p-mTOR in high glucose–cultured podocytes transfected with scramble or Rheb1 siRNA for 24 hours. (**D** and **E**) The cultured podocytes were pretransfected with scramble or Rheb1 siRNA for 24 hours, or treated with rapamycin for 30 minutes, followed by high glucose treatment for 24 hours. Propidium iodide (PI) staining (**D**) and quantitative analysis (**E**) of dead cells among different groups. Data are presented as the percentage of PI-staining-positive cells. ^#^*P* < 0.05 vs. scramble control cells, *n* = 3; *P < 0.05, ^$^*P* < 0.05 vs. high glucose–treated podocytes, *n* = 3. Scale bar: 20 μm. (**F**) UACR in Podo-Rheb1^+/+^ and Podo-Rheb1^–/–^ mice at 6 months after STZ-induced DM. *P < 0.05 vs. Podo-Rheb1^+/+^ mice, *n* = 6–7. (**G**) Representative PAS staining and immunofluorescence staining for diabetic kidney injury, WT1, and nephrin among different groups. Scale bar: 50 μm. (**H**–**J**) Quantitative analyses of injury score, glomerular area, and WT1-positive podocytes per glomerulus among different groups. ^#^*P* < 0.05 vs. vehicle control mice; *P < 0.05 vs. Podo-Rheb1^+/+^ mice with STZ injection, *n* = 3–7. Data are expressed as the mean ± SEM. Comparison between the groups was performed using 1-way ANOVA followed by the Tukey test (**B** and **E**). Comparison between the groups was performed using the 2-tailed Student’s *t* test (paired *t* test) (**F**). Data in **H**–**J** were analyzed with 2-way ANOVA with Tukey’s post hoc test.

In contrast to the mechanistic understanding of how mTORC1 senses amino acids (33, 42–44), much less is known about how mTORC1 senses glucose. The above data showed that Rheb1 mediates Tsc1 ablation–stimulated mTORC1 activation. Whether Rheb1 mediates high glucose–induced mTORC1 activation in podocytes is not clear. To explore this hypothesis, we first examined the abundance and activity of Rheb1 in podocytes in response to high glucose treatment. The results demonstrated that high glucose did not obviously increase the abundance or activity of Rheb1 (Figure 6, A and B). Furthermore, high glucose–induced mTORC1 activation was not inhibited by Rheb1 knockdown (Figure 6C), suggesting a dispensable role for Rheb1 in high glucose–induced mTORC1 activation in podocytes. Interestingly, rapamycin treatment protected against glucose-induced podocyte death, while Rheb1 knockdown aggravated high glucose–induced podocyte death (Figure 6, D and E), suggesting that Rheb1 may promote podocyte survival independent of mTORC1 signaling. Interestingly, in mice with podocyte-specific Rheb1 gene deletion, more severe albuminuria and diabetic kidney injury developed compared with those in Podo-Rheb1^+/+^ mice after STZ administration (Figure 6, F–J). Therefore, the data above indicate that high glucose–activated mTORC1 signaling is independent of Rheb1 in podocytes.

### PP2Acα deficiency mediates high glucose–stimulated mTORC1/STAT1/CFB activation and podocyte injury.

**Figure 7:**
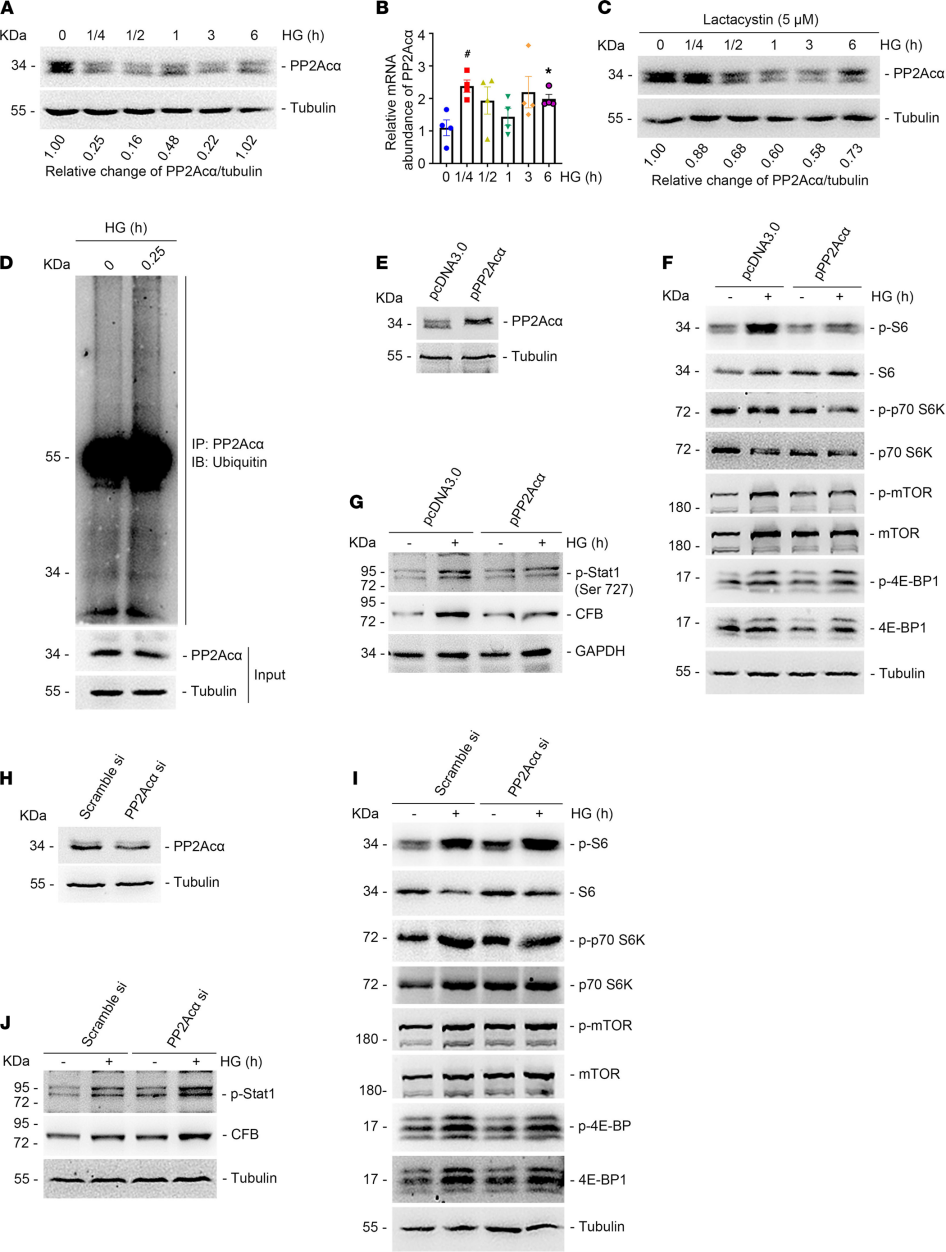
PP2Acα deficiency mediates high glucose–induced mTORC1 activation and CFB upregulation in podocytes. (**A**) Western blot assay showing the abundance of PP2Acα in cultured podocytes after high glucose treatment at different times. Relative change of PP2Acα/tubulin shown at the bottom of the bands. (**B**) Real-time PCR analysis showing the mRNA abundance for PP2Acα in cultured podocytes after high glucose treatment. *P < 0.05 and ^#^*P* < 0.05 vs. control cells, *n* = 4. (**C**) Western blot assay showing the abundance of PP2Acα in cultured podocytes pretreated with lactacystin for 30 minutes, followed by high glucose treatment at different times. Relative change of PP2Acα/tubulin shown under the bands. (**D**) The cultured podocytes were pretreated with lactacystin for 30 minutes, followed by high glucose treatment for 15 minutes. Western blot assay showing the level of ubiquitin in the precipitates (PP2Acα-IP) and PP2Acα in lysates (input). (**E**) Western blotting assay showing the expression of PP2Acα (MR204384) in cultured podocytes after PP2Acα (MR204384) plasmid transfection. (**F** and **G**) Western blotting assays showing the abundance of p-S6, p-p70 S6K, p-4E-BP1, p-mTOR (**F**), p-Stat1 (Ser727), and CFB (**G**) in high glucose–cultured podocytes transfected with pcDNA3.0 or PP2Acα (MR204384) plasmid for 36 hours. (**H**) Western blotting analyses demonstrating the downregulation of PP2Acα after PP2Acα siRNA transfection. (**I** and **J**) Western blotting assays showing the abundance of p-S6, p-p70 S6K, p-4E-BP1, p-mTOR (**I**), p-Stat1 (Ser727), and CFB (**J**) in high glucose–cultured podocytes transfected with scramble or PP2Acα siRNA for 36 hours. Data are expressed as the mean ± SEM. Comparison between the groups was performed using 1-way ANOVA followed by the Tukey test.

Protein phosphatase 2A (PP2A), a primary serine/threonine phosphatase, is ubiquitously expressed in eukaryotic cells (45). Recent studies have reported that PP2A may protect against podocyte injury in DKD (46, 47). Therefore, we speculated that PP2A contributes to podocyte injury and DKD by modulating mTORC1/CFB/complement alternative pathway activation. To explore this, we first treated podocytes with high glucose, and the results showed that PP2Acα protein abundance was largely decreased from 15 minutes to 3 hours after high glucose treatment (Figure 7A), whereas mRNA levels of PP2Acα were increased during the observation period (Figure 7B), suggesting that PP2Acα downregulation may have occurred at the posttranscriptional level. We then treated podocytes with lactacystin, a proteasomal inhibitor. The results showed that lactacystin largely abolished high glucose–induced PP2Acα degradation (Figure 7C). Additionally, PP2Acα ubiquitination was markedly increased after high glucose treatment in podocytes (Figure 7D), suggesting that high glucose may promote PP2Acα degradation through the proteasome pathway.

We next overexpressed or knocked down PP2Acα expression in podocytes using the PP2Acα plasmid or PP2Acα siRNA transfection, respectively. Western blot assays showed that overexpression of PP2Acα reduced high glucose–induced mTORC1 activation and p-STAT1 and CFB abundance in cultured podocytes (Figure 7, E–G). In contrast, PP2Acα knockdown enhanced mTORC1 activation and p-STAT1 and CFB induction in podocytes cultured with high glucose (Figure 7, H–J), suggesting that PP2Acα plays an essential role in mediating high glucose–stimulated mTORC1 signaling activation.

To further investigate the role of PP2Acα in podocyte injury in DKD, we generated mice with podocyte-specific PP2Acα gene deletion using the Cre-*LoxP* system (Supplemental Figure 4A). Immunofluorescence staining images revealed a reduction in PP2Acα in podocytes from Podo-PP2Acα^–/–^ mice compared with control littermates (Supplemental Figure 4B). Mice were born normal, and there was no obvious difference in urinary glucose levels, UACR, blood glucose, levels of blood urea nitrogen (BUN), body weight, kidney weight, kidney/body weight ratio, or histological structure of the kidney between Podo-PP2Acα^–/–^ mice and Podo-PP2Acα^+/+^ mice (Supplemental Figure 4, C–J). Next, we injected STZ into these mice to induce DKD (Supplemental Figure 4K). Podo-PP2Acα^–/–^ mice developed severe albuminuria and podocyte injury compared with their control littermates after STZ administration (Supplemental Figure 4, L and M). Obvious induction of p-S6, p-STAT1, CFB, C3d, and C5b-9 was observed in podocytes from Podo-PP2Acα^–/–^ mice administered STZ (Supplemental Figure 4M). Therefore, these data indicate that PP2Acα deficiency contributes to high glucose–induced mTORC1 activation, CFB induction, and alternative complement pathway activation, which aggravates DKD in mice.

## Discussion

Here, we reported that high glucose stimulates CFB upregulation and alternative complement pathway activation, which in turn promotes podocyte injury and DKD. In addition, we demonstrated that PP2Acα/mTORC1/STAT1 signaling mediates high glucose–stimulated CFB upregulation, complement alternative pathway activation, and podocyte injury. To our knowledge, this is the first study to identify a mechanism for stimulating alternative complement pathway activation in podocytes in response to high glucose levels in DKD.

The complement system is an extremely effective cell-killing and inflammation-provoking pathway. It is controlled strictly by different inhibitory mechanisms to prevent excessive activation. Major complement-related diseases are associated with overwhelming complement activation because of an abnormal inhibition system of complement activation. Notably, the kidney is vulnerable to complement attack, especially due to inappropriate alternative complement pathway activation (48, 49). In addition to common complement-mediated kidney diseases, such as lupus nephritis, atypical hemolytic uremic syndrome, C3 glomerulopathies, ANCA-associated GN, and IgA nephropathy, previous studies have revealed that the complement system is activated in DKD as determined by analysis of urinary proteins and histological components in kidney biopsies. Marikanty et al. found that the majority of urinary proteins from DKD patients were enriched with alternative complement and blood coagulation pathways (50). Zhao et al. discovered that the higher levels of various complement components in plasma and urine and the deposition of C1q and C3c in renal tissues are highly correlated with proteinuria and glomerular lesions in DKD patients (27, 28). Consistently, by analyzing GSE30528 data uploaded, using KEGG and GO analysis, we found that genes in complement system activation were enriched in DKD kidney biopsies (34). Immunohistochemical staining confirmed the remarkable elevation of CFB, C3d, C5aR, and C5b-9 abundance in DKD glomeruli, and a markedly positive correlation between CFB induction in podocytes and glomerular enlargement was observed.

Although the main source of circulating complement is the liver, smaller but significant amounts of complement components, including CFB, are produced in other organs (kidney, brain, blood vessels, lungs, intestine, joints, and skin) (51). Grossman et al. confirmed that a reduction in circulating factor B levels, achieved by antisense oligonucleotides, significantly improved renal pathology and reduced glomerular C3 deposition and proteinuria in mice with lupus nephritis (13). Chen et al. found that upregulation of CFB in retinal pigment epithelial cells is accompanied by complement activation in the aged retina (52). Zhang et al. uncovered that mutation of CFB led to disordered activation of the liquid phase alternative pathway, which increased the deposition of C3b on the cell surface and contributed to the occurrence of atypical hemolytic uremic syndrome (15). In DKD mouse models, we found that downregulation of CFB using CFB shRNA largely inhibited renal alternative complement pathway activation and ameliorated diabetic podocyte injury and dysfunction. Our findings indicate that activation of the alternative complement pathway by local CFB synthesis in kidney contributes to podocyte injury and DKD. However, due to enrichment of CFB shRNA in host liver, circulating CFB synthesized by liver cells is first reduced. Therefore, we cannot rule out the effect of systemic CFB reduction on podocyte injury and DKD.

An important question remaining is how the alternative complement pathway is activated in DKD kidneys. Again, based on the transcriptome analysis of human DKD reported (34), we found that genes were enriched in the mTORC1 signaling pathway in DKD glomeruli. In patients with DKD and animal models, mTORC1 signaling was activated, and blockade of mTORC1 signaling with rapamycin abolished alternative complement pathway activation in podocytes in a DKD animal model. In addition, activation of podocyte mTORC1 signaling upregulated CFB expression and stimulated alternative complement pathway activation. Wu et al. reported that STAT1 may upregulate CFB expression in ADPKD (41). In this study, we found that activation of mTORC1 signaling led to STAT1 phosphorylation, which in turn upregulated CFB expression. Therefore, we concluded that mTORC1/STAT1 signaling activation stimulates CFB upregulation and alternative complement pathway activation to induce podocyte dysfunction and DKD.

mTORC1 activation is tightly controlled by numerous upstream inputs, including glucose, amino acids, and growth factors, via various sensing pathways (53). To identify the connection between high glucose and complement alternative pathway activation in DKD, in this study, we further explored the sensing pathway of high glucose–induced mTORC1 activation. First, we found that neither Rheb1 abundance nor its activity was affected by high glucose treatment. Knocking down Rheb1 gene expression did not weaken the capacity of high glucose to activate mTORC1 in podocytes. Furthermore, Rheb1 deletion in podocytes even aggravated podocyte injury in STZ-induced DKD mice. Therefore, these data preclude the possibility that Rheb1 mediates high glucose–stimulated mTORC1 signaling activation in podocytes. Additionally, we showed that PP2Acα contributes to podocyte injury and DKD, which was supported by Zhong et al.’s report that podocyte-specific loss of PP2A enhanced podocyte injury and loss, suggesting that PP2A has a protective role in podocyte injury in DKD (47). Several lines of evidence in our study demonstrated that PP2Acα deficiency promotes podocyte injury by modulating mTORC1/CFB/complement alternative pathway activation. First, high glucose treatment reduced PP2Acα protein abundance from 15 minutes to 3 hours after treatment in podocytes. Second, in cultured podocytes, overexpression of PP2Acα reduced, while knocking down PP2Acα expression enhanced, high glucose–induced mTORC1 activation and CFB abundance. Third, in mice with podocyte PP2Acα gene deletion, obvious induction of p-S6, p-STAT1, CFB, C3d, and C5b-9 was detected in podocytes from Podo-PP2Acα^–/–^ mice with STZ administration. Interestingly, we found that high glucose may promote PP2Acα degradation through the proteasome pathway, which mediates mTORC1 activation induced by high glucose and further activates complement alternative pathway activation. However, further work is still needed to clarify how high glucose affects PP2Acα protein stability in podocytes.

In summary, this study revealed that mTORC1/STAT1 signaling activation mediates high glucose–induced CFB upregulation and alternative complement activation to promote podocyte injury and DKD. Additionally, we demonstrated that PP2Acα degradation but not Rheb1 plays an important role in enhancing high glucose- and diabetes-induced mTORC1 activation in podocytes.

## Methods

### Study design.

In this study, we aimed to explore the mechanism of complement alternative pathway activation in DKD. According to GSE30528 data, we focused on the mTORC1 signaling pathway. Human kidney samples with fewer than 5 glomeruli were excluded. In in vivo studies, we generated 2 types of DM mice, and investigators performing the surgeries were blinded to the experimental conditions. Researchers imaging and collecting data were unaware of which animals represented which groups, but data were not analyzed in a blinded manner.

### Mice.

Homozygous *Tsc1*-floxed mice (005680, The Jackson Laboratory) and podocin-Cre transgenic mice (008205, The Jackson Laboratory) were ordered. *Rheb1*-floxed mice (C57BL/6J background) were provided by Bo Xiao (Southern University of Science and Technology, Guangzhou, China) (54, 55). Homozygous *PP2Acα*-floxed mice (C57BL/6J background) were provided by Chaojun Li (Nanjing University, Nanjing, China) (56). Podocyte-specific Tsc1, Rheb1, Tsc1 plus Rheb1 or PP2Acα knockout mice (genotypes: Podo-Cre^+/–^, Tsc1^fl/fl^; Podo-Cre^+/–^, Rheb^fl/fl^; Podo-Cre^+/–^, Tsc1^fl/fl^, Rheb^fl/fl^; Podo-Cre^+/–^, PP2Acα^fl/fl^) were generated by crossbreeding podocin-Cre transgenic mice with TSC1-, Rheb1- or PP2Acα-floxed mice. Litters of the same sex with genotypes Podo-Cre^–/–^, Tsc1^fl/fl^; Podo-Cre^–/–^, Rheb^fl/fl^; and Podo-Cre^–/–^, PP2Acα^fl/fl^ were considered littermate controls. Genotyping was performed by PCR using DNA extracted from mouse tails. The primers used for genotyping were as follows: *Cre* transgene, sense: 5*′*-CTGATTTCGACCAGGTTCGT-3*′* and antisense: 5*′*-ATTCTCCCACCGTCAGTACG-3*′*; *Tsc1* gene, sense: 5*′*-GTCACGACCGTAGGAGAAGC-3*′* and antisense: 5*′*-GAATCAACCCCACAGAGCAT-3*′*; *Rheb1* gene, sense: 5*′*-GCCCAGAACATCTGTTCCAT-3*′* and antisense: 5*′*-GGTACCCACAACCTGACACC-3*′*; *PP2Acα* gene, sense: 5*′*- AAGTTACTGAGTGCAGTGTGCCTTG-3*′* and antisense: 5*′*-TTATACCCTTCCTCATTCGCTCTGC-3*′*. All animals were born normal with the expected Mendelian frequency. All animals were maintained in the specific pathogen-free (SPF) Laboratory Animal Center of Nanjing Medical University according to the guidelines of the Institutional Animal Care and Use Committee of Nanjing Medical University. Male CD-1 mice (18–20 g) were acquired from the SPC Laboratory Animal Center of Nanjing Medical University. Male db/db mice (3 weeks old) were ordered from GemPharmatech.

### Animal models.

Mice were subjected to right renal nephrectomy. Two days later, these mice were intraperitoneally injected with STZ dissolved in citrate buffer at 50 mg/kg for 4 consecutive days. One week after the last STZ injection, mice with blood glucose greater than 16.7 mmol/L were included in the experiments. All male db/db mice developed high blood glucose (>16.7 mmol/L) at 4 weeks old. For rapamycin treatment, mice were intraperitoneally injected with 1 mg/kg rapamycin every other day for 5 weeks. Urine samples were collected at 3 and 6 months for urinary albumin excretion, and the mice were euthanized at 6 months after the onset of DM. Serum, urine, and kidney tissue were analyzed for biochemical parameters and renal histology.

### Functional and pathway enrichment analysis of the microarray data set.

GSE30528 was downloaded from the Gene Expression Omnibus and analyzed with GEO2R. A total of 22 samples were used in this data set, including 9 DKD and 13 control samples. The GO project provides a controlled vocabulary to describe gene and gene product attributes in any organism. Fisher’s exact test was used to determine whether there was more overlap between the differential expression list and the GO annotation list than would be expected by chance. KEGG is a knowledge base for the systematic analysis of gene functions in terms of networks of genes and molecules. In this study, GO and KEGG pathway enrichment analyses of differentially expressed genes were performed via DAVID, https://david.ncifcrf.gov/, and KOBAS, http://kobas.cbi.pku.edu.cn/anno_iden.php Then, combined with the results of GEO2R analysis (*P* < 0.05), genes enriched in mTORC1 signaling or complement and coagulation cascades were shown using a heatmap.

### Urinary albumin and creatinine assay.

Urinary albumin levels were measured using a mouse albumin ELISA quantification kit according to the manufacturer’s protocol (Bethyl Laboratories). Urinary creatinine levels were determined using a QuantiChrom Creatinine Assay kit according to the protocol (DICT-500, BioAssay System). Urinary proteins were also analyzed using SDS-PAGE after normalization to urinary creatinine.

### SCr and BUN assay.

SCr was measured using the QuantiChrom Creatinine Assay kit (DICT-500), and BUN in serum was measured using the QuantiChrom Urea Assay kit (DIUR-500) according to the manufacturer’s instructions.

### Plasmids.

Short hairpin RNA specific for the mouse CFB gene was ordered from Ruizhen. The target sequence of the murine CFB is GAATTCGATAAGAGGGTACCAGCTGTTGGTTTTGGCCACTGACTGACCAACAGCTTACCCTCTTATCACCGG. The target sequence of the scrambled shRNA is AAATGTACTGCGCGTGGAGAC. The mouse CFB-specific shRNA was subcloned into pcDNA6.2. STZ-induced DM mice and db/db mice were injected with 1 mg/kg CFB shRNA and pcDNA6.2 through the tail vein every week 4 times, followed by every 2 weeks for 5 months, to induce CFB downregulation.

### Cell culture and treatment.

The conditionally immortalized mouse podocyte cell line was provided by Peter Mundel (Mount Sinai School of Medicine, New York, New York, USA) and was previously described (57). Cells were cultured at 33°C in RPMI-1640 medium (Gibco, Thermo Fisher Scientific) supplemented with 10% fetal bovine serum and recombinant interferon-γ (Invitrogen, Thermo Fisher Scientific). To induce differentiation, podocytes were grown under nonpermissive conditions at 37°C in the absence of interferon-γ. After serum and glucose starvation for at least 12 hours, cells were treated with high glucose (30 mM) and mTOR activator for various periods of time as indicated. In some experiments, cells were pretreated with rapamycin, fludarabine, or lactacystin for 30 minutes before incubation with stimuli. Rheb1, PP2Acα, or scramble siRNA (IBSBIO) and pcDNA3.0 or pCMV6-Myc-DDK–tagged PP2Acα plasmid (MR20438, OriGen) were transfected into podocytes using Lipofectamine 3000 reagent (Invitrogen, Thermo Fisher Scientific) according to the manufacturer’s instructions. Further cell culture details can be found in Supplemental Methods.

### Histology and immunohistochemistry.

Paraffin-embedded kidney sections (3 μm thickness) were stained using PAS. A semiquantitative scoring method was used to define kidney injury by the loss of brush border, tubular cell necrosis, and cellular casts. A score of 0 represents an injury area less than 10%, whereas scores of 1, 2, 3, and 4 represent injuries involving 10%–25%, 25%–50%, 50%–75%, and more than 75% of the kidney tissue, respectively. At least 5 randomly selected fields under a ×400 original magnification microscope were evaluated for each mouse, and an average score was calculated. Assessment of the glomerular cross-sectional areas was performed using pixel counts on the kidney section in a blinded fashion under 400× original magnification (OLYMPUS DP74 and BX53 epifluorescence microscope equipped with a digital camera).

For immunohistochemical staining, paraffin-embedded kidney tissue sections were deparaffinized and hydrated. Heat-induced epitope retrieval was employed. Endogenous peroxidase activity was quenched by 3% H_2_O_2_. Tissue sections were then blocked in 10% normal donkey serum, followed by incubation with anti-p-S6 (catalog 4858, Cell Signaling Technology), anti-Rheb1 (catalog ab25873, Abcam), anti-CFB (catalog ab192577, Abcam), anti-C3d (catalog AF2655, R&D Systems, Bio-Techne), anti-C5b-9 (catalog ab55811, Abcam), anti-C5aR (catalog BS1522, Bioworld), anti–p-STAT1 (Ser727) (catalog 8826S, Cell Signaling Technology), and anti–p-STAT1 (Tyr701) (catalog 7649S, Cell Signaling Technology) overnight at 4°C. After incubation with secondary antibody (anti–mouse IgG: BA-9200, Vector Laboratories; anti–rabbit IgG: BA-1000, Vector Laboratories; anti–goat IgG: BA-9500, Vector Laboratories) for 1 hour, sections were incubated with ABC reagents for 1 hour at room temperature before being subjected to substrate 3-amino-9-ethylcarbazole for staining (Vector Laboratories). Slides were viewed with an OLYMPUS DP74 and BX53 epifluorescence microscope equipped with a digital camera. Ten glomeruli were selected from each slide, and then we counted the total number of CFB/C3d/C5b-9/C5aR-positive podocytes, finally calculating the average number of CFB-positive podocytes in each glomerulus.

### Immunofluorescence staining.

Kidneys were frozen in Optimum Cutting Temperature compound (Sakura) and sectioned at 3 μm thickness (Leica Kryostat). The sections were fixed in 4% paraformaldehyde for 15 minutes, permeabilized in 0.5% Triton X-100 in PBS for 5 minutes at room temperature, and then blocked in 2% normal donkey serum for 60 minutes. The sections were then immunostained with the following antibodies: anti-WT1 (catalog BS91456, Bioworld), anti-nephrin (Progen), anti-PP2Acα (catalog ab106262, Abcam), and anti–p-s6 (catalog 4858, Cell Signaling Technology).

The cultured podocytes seeded on coverslips were fixed in cold methanol/acetone (1:1) for 10 minutes at –20°C. After 3 extensive washes with 1× PBS, cells were treated with 1% Triton X-100 for 5 minutes, blocked in 2% normal donkey serum in 1× PBS buffer for 40 minutes at room temperature, and incubated with the following antibodies: anti–p-s6 (catalog 4858, Cell Signaling Technology), anti-CFB (catalog ab192577, Abcam), and anti–p-STAT1 (Ser727) (catalog 8826S, Cell Signaling Technology), followed by staining with FITC- or tetramethylrhodamine-conjugated secondary antibodies: Cy3-AffiniPure Donkey Anti-Rabbit IgG (H+L): 711-165-152, Jackson ImmunoResearch. Cells were also stained with DAPI to visualize the nuclei. Slides were viewed using an OLYMPUS DP74 and BX53 epifluorescence microscope equipped with a digital camera.

### TEM.

The kidney sections were fixed in 3.7% glutaraldehyde in PBS buffer. After rinsing and postfixing in 1% osmium tetroxide, samples were embedded in 10% gelatin, fixed, and cut into several blocks (<1 mm^3^). After dehydration in increasing concentrations of alcohol and infiltration with increasing concentrations of Quetol-812 epoxy resin mixed with propylene oxide, samples were embedded in pure, fresh Quetol-812 epoxy resin and polymerized. Ultrathin sections (100 nm) were cut using a Leica UC6 ultramicrotome and poststained with uranyl acetate for 10 minutes and lead citrate for 5 minutes at room temperature before observation under a transmission electron microscope (JEOL JEM-1010).

### Measurement of Rheb1 activation in cultured podocytes.

The cultured podocytes were treated with 30 mM glucose for 0.25, 1, and 3 hours as indicated. Cells were harvested to measure Rheb1 activation according to a previous report (55).

### Immunoprecipitation.

The cultured podocytes were pretreated with lactacystin for 30 minutes, followed by high glucose treatment for 15 minutes. Cells were harvested after being washed 2 times with cold PBS and extracted in immunoprecipitation buffer (P0013, Beyotime) containing 0.5% PMSF, 1% protease inhibitor cocktail (HY-K0010, MedChemExpress), and 1% phosphatase I (HY-K0021, MedChemExpress), II (HY-K0022, MedChemExpress), and III (HY-K0023, MedChemExpress) inhibitor cocktails. Lysates (1 mg) were incubated with anti-PP2Acα (catalog ab106262, Abcam) at 4°C overnight. Protein A/G agarose beads were then incubated with the lysates for 1 hour at 4°C. Beads were washed with lysis buffer and PBS, resuspended in 35 μL of 2× SDS buffer, and boiled for 5 minutes. Samples were subjected to SDS-PAGE. Western blotting was performed by standard protocols using ECL reagents (Vazyme).

### Western blot analysis.

The cultured podocytes were harvested with 1× SDS sample buffer. The kidneys were lysed in RIPA solution containing 1% NP-40, 0.1% SDS, 100 mg/mL PMSF, 1% phosphatase I and II inhibitor cocktail, and 1% protease inhibitor cocktail (MilliporeSigma) on ice for 30 minutes. The supernatants were collected after centrifugation at 16,000*g* at 4°C for 30 minutes. Protein concentration was determined using the bicinchoninic acid protein assay (BCA kit; Pierce, Thermo Fisher Scientific) according to the manufacturer’s instructions. An equal amount of protein was loaded into 10% or 15% SDS-PAGE gels and transferred onto PVDF membranes. The primary antibodies were as follows: anti–p-S6 (catalog 4858, Cell Signaling Technology), anti-S6 (catalog 2217, Cell Signaling Technology), anti-Rheb1 (catalog ab25873, Abcam), anti–p-p70 S6K (Thr421/Ser424) (catalog 9204L, Cell Signaling Technology), anti-p70 S6K (catalog 9202, Cell Signaling Technology), anti–p-4E-BP (Thr37/46) (catalog 2855, Cell Signaling Technology), anti–4E-BP (catalog 9452, Cell Signaling Technology), anti–p-mTOR (catalog 5536, Cell Signaling Technology), anti-mTOR (catalog 2983, Cell Signaling Technology), anti-PP2Acα (catalog ab106262, Abcam), anti-CFB (catalog ab192577, Abcam), anti–p-STAT1 (Ser727) (catalog 8826S, Cell Signaling Technology) anti–p-STAT1 (Tyr701) (catalog 7649S, Cell Signaling Technology), anti-ubiquitin (catalog sc-8017, Santa Cruz Biotechnology), anti–β-actin (catalog sc-47778, Santa Cruz Biotechnology), and anti-GAPDH (catalog AP0063, Bioworld). Quantification was performed by measuring the signal intensity with the aid of NIH ImageJ software package.

### Quantitative real-time PCR.

For determination of PP2Acα mRNA expression, semiquantitative reverse transcriptase PCR was used. Total RNA was extracted using TRIzol reagent (Invitrogen, Thermo Fisher Scientific) according to the manufacturer’s instructions. cDNA was synthesized with 1 μg of total RNA, ReverTra Ace (Vazyme), and oligo (dT) 12–18 primers. A quantitative real-time PCR assay was used to quantitate the relative PP2Acα mRNA abundance using a Light Cycler 96 System (Roche). The relative amount of mRNA versus the internal control was calculated using the equation 2ΔCT, in which ΔCT = CT^gene^ – CT^control^.

### Data and materials availability.

Microarray data are available in the Gene Expression Omnibus curated by the National Center for Biotechnology Information under the accession number GSE30528.

### Statistics.

The data are expressed as scatter plots with bar (mean ± SEM). Western blot analysis was completed by scanning and analyzing the intensity of hybridization signals using the NIH ImageJ program. Statistical analysis of the data was performed using GraphPad Prism 8 (GraphPad Software). We used Pearson’s correlation to determine relationships between variables. For the Rheb1-knockout study, data were analyzed with 2-way ANOVA with Tukey’s post hoc test.

We performed between-group comparisons using the 2-tailed Student’s *t* test. *P* < 0.05 was considered statistically significant.

### Study approval.

Human kidney specimens diagnosed with DKD by independent pathologists were obtained from diagnostic renal biopsies performed at the Second Affiliated Hospital of Nanjing Medical University. The adjacent healthy kidney tissues from patients with kidney tumors from the Second Affiliated Hospital of Nanjing Medical University were used as controls. General DKD patient information is shown in Supplemental Table 1. The mean age ± SD was 60.5 ± 6.83 years for controls (3 men and 3 women). The Institutional Review Board at the Second Affiliated Hospital approved all studies involving human tissues and waived the requirement for informed consent.

## Author contributions

QL and CD conceptualized the study and developed the methodology. QL, QH, KC, YL, XS, MG, and XX performed experiments. QL and CD analyzed and interpreted data. QL drafted the paper. AZZ and CD reviewed and edited the paper. QL coordinated and managed experiments. CD supervised the study.

## Supplementary Material

Supplemental data

## Version 1 10/08/2021

Electronic publication
