# Supplementary material for: Complement factor B in high glucose–induced podocyte injury and diabetic kidney disease
Source: JCI Insight. 2021 Oct 8;6(19):e147716. doi: 10.1172/jci.insight.147716 (PMC8525650; doi:10.1172/jci.insight.147716)
Supplement: Supplemental data [file jciinsight-6-147716-s049.pdf]

Supplementary Materials

Cell culture and treatment. Normal rat kidney tubular epithelial cells (NRK-52E) were ordered from ATCC (Manassas, VA) and cultured in Dulbecco's modified Eagle's medium-F12 medium supplemented with 5% fetal bovine serum (Invitrogen, Grand Island, NY). Primary tubular epithelial cells were isolated from the kidneys of mice (one week old) and cultured in Dulbecco's modified Eagle's medium-F12 medium supplemented with 10% fetal bovine serum (Invitrogen, Grand Island, NY).

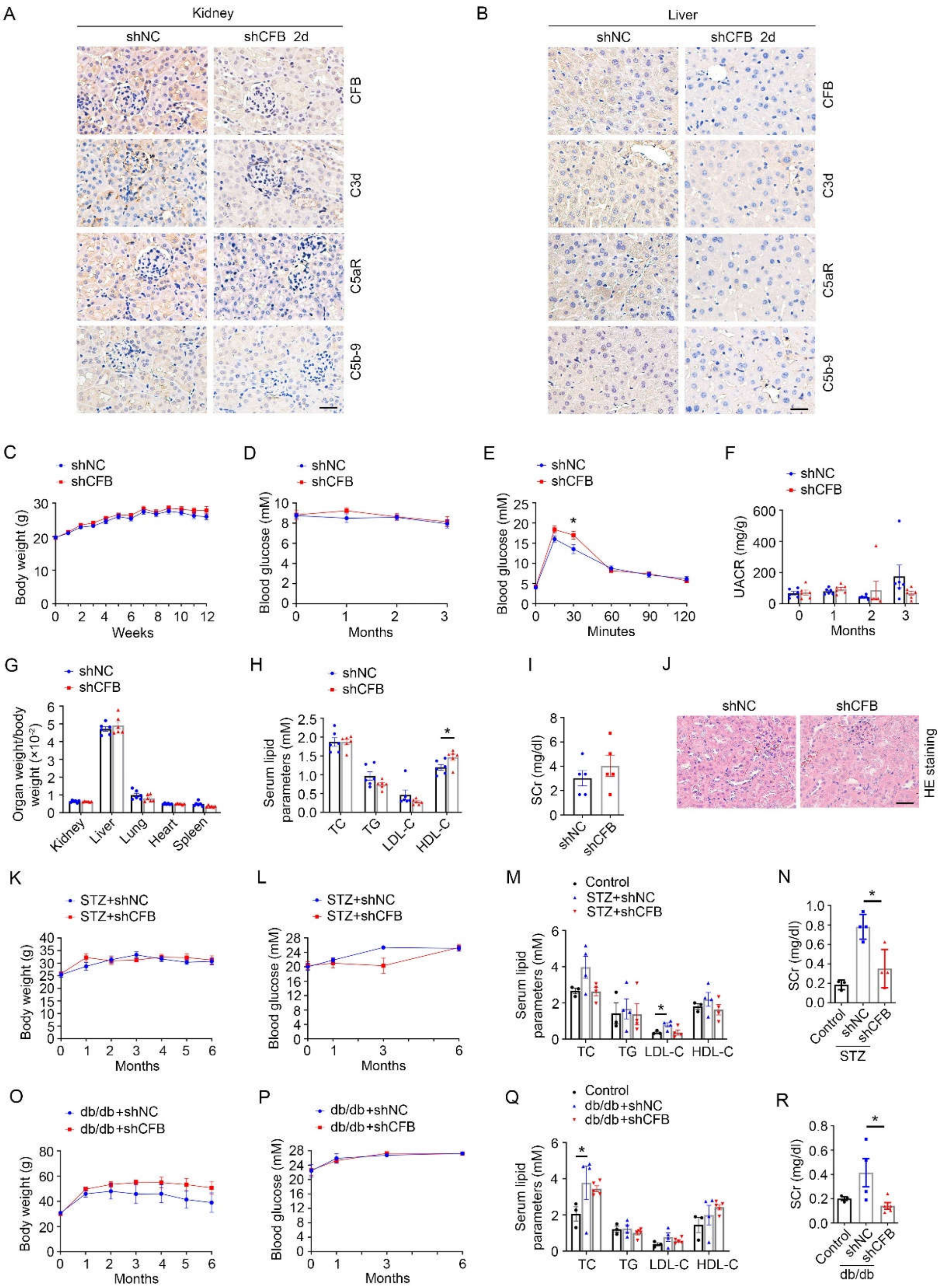

Figure S1. The role of CFB knockdown in mice under normal and high glucose conditions. (A, B) Representative immunohistochemical staining images for CFB, C3d, C5b-9 and C5aR in glomerulus (A) and liver (B) at day 0, 2 and 7 after CFB shRNA injection. Scale bar =20 μm. (C-I) The graphs showing body weight (C), blood glucose (D), intraperitoneal glucose tolerance test (IPGTT) (E), UACR (F), organ /body weight ratio (G), serum lipid parameters (H), the level of serum creatinine (I) between the control mice injected with scramble shRNA and CFB shRNA. *P<0.05 vs shNC, n=5. (J) Kidney histology as shown by hematoxylin-eosin (HE) staining. Scale bar = 20 μm.

(K-N) The graphs showing body weight (K), blood glucose (L), serum lipid parameters (M) and the level of serum creatinine (N) among different groups. *P<0.05 vs shNC, n=4. (O-R) The graphs showing body weight (O), blood glucose (P), serum lipid parameters (Q) and the level of serum creatinine (R) among different groups. *P<0.05 vs shNC, n=4. Data are expressed as the mean ± SEM. Comparison between the groups was performed using the two-tailed Student t test (unpaired t test).

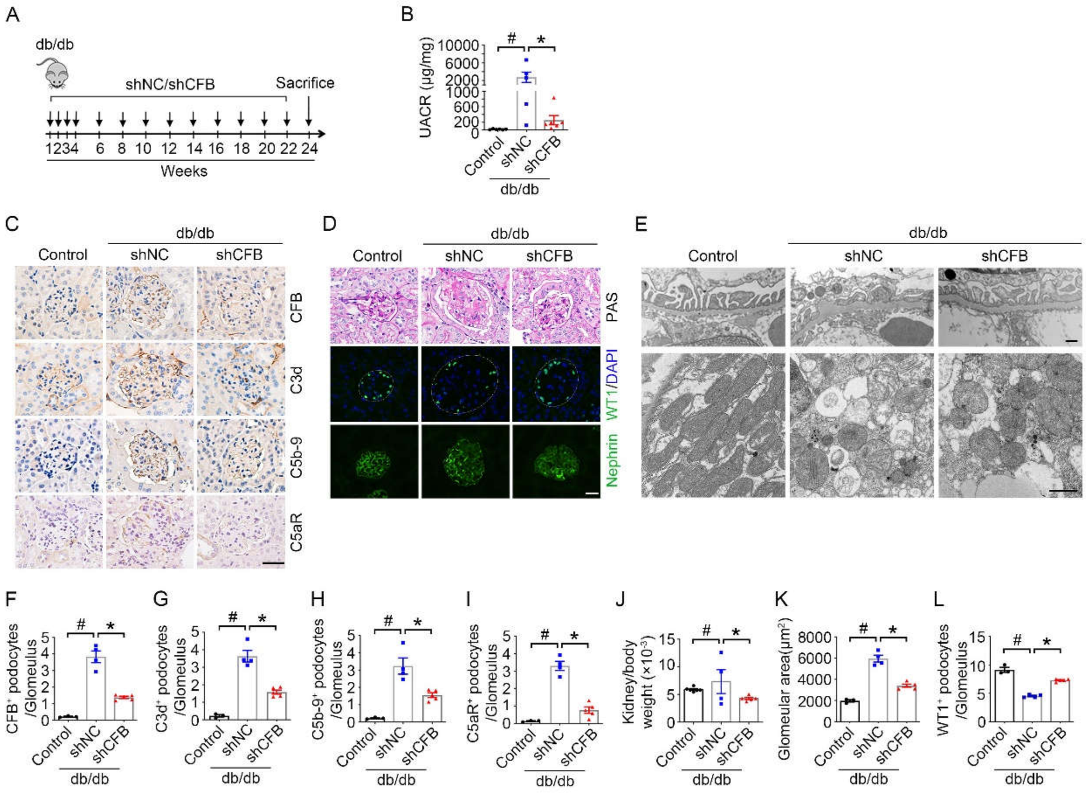

Figure S2. CFB knockdown attenuates podocyte injury and DKD in db/db mice. (A) The strategy for db/db mice injected with CFB shRNA (shCFB) via tail vein. (B) Urinary albumin levels among different groups. #P<0.05, *P<0.05, n=5-6. (C) Representative immunohistochemical staining images showing the induction of CFB, C3d, C5b-9 and C5aR in glomerulus among different groups. Scale bar = 20 μm. (D) Representative PAS staining and representative immunofluorescent staining images for diabetic kidney injury, WT1 and nephrin among different groups. Scale bar = 20 μm. (E) Representative TEM images. Scale bar = 400 nm (above). Scale bar =1μm (below). (F-I) Quantitative analyses of CFB, C3d, C5b-9 and C5aR-staining positive podocytes per glomerulus among different groups. #P<0.05, *P<0.05, n=3-5. (J) The graphs showing the kidney/body weight ratio among different groups. #P<0.05, *P<0.05, n=4-6. (K, L) Quantitative analyses of glomerular area (K) and WT1 positive podocytes per glomerulus (L) among different groups. #P<0.05, *P<0.05, n=3-5. Data are expressed as the mean ± SEM. Comparison between the groups was performed using one-way ANOVA followed by the Tukey test.

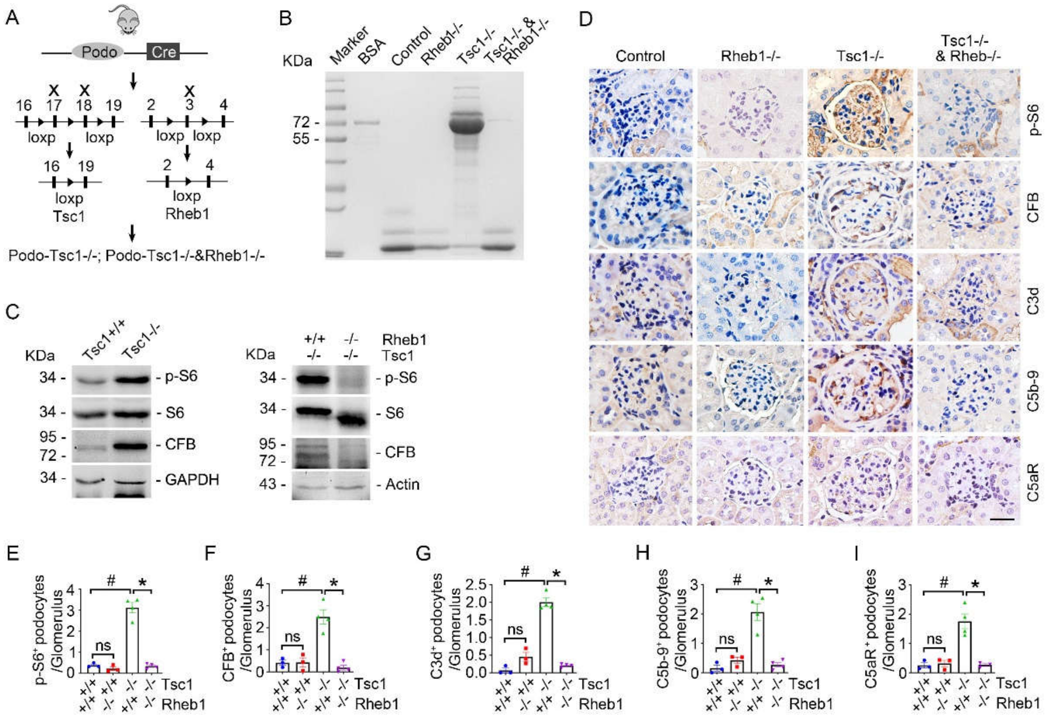

Figure S3. Ablation of Rheb1 downregulates mTORC1-induced CFB expression in podocytes and prevents podocyte injury. (A) Schematic of the recombination strategy for generating mice with Tsc1, Tsc1 plus Rheb1 gene deletion in podocytes. (B) Representative sodium dodecyl sulfate–polyacrylamine gelelectrophoresis showing the urinary albumin after normalization to urinary creatinine (Ucr) in control, podo-Rheb1-/-, podo-Tsc1-/- and podo-Tsc1 -/-&Rheb1-/- mice. Urine proteins after separation with SDS-PAGE gel were stained with Coomassie blue R-250. BSA, bovine serum albumin. (C) Western blot assay showing the induction of p-S6 and CFB in mouse kidneys with podocyte Tsc1 gene deletion or Tsc1 plus Rheb1 gene deletion. (D) Representative immunohistochemical staining for p-S6, CFB, C3d, C5b-9 and C5aR in glomerulus among different groups. Scale bar =20 μm. (E-I) Quantitative analyses of p-S6, CFB, C3d, C5b-9 and C5aR positive podocytes per glomerulus among different groups. #P<0.05, *P<0.05, n=4-7. Data are expressed as the mean ± SEM. Comparison between the groups was performed using one-way ANOVA followed by the Tukey test.

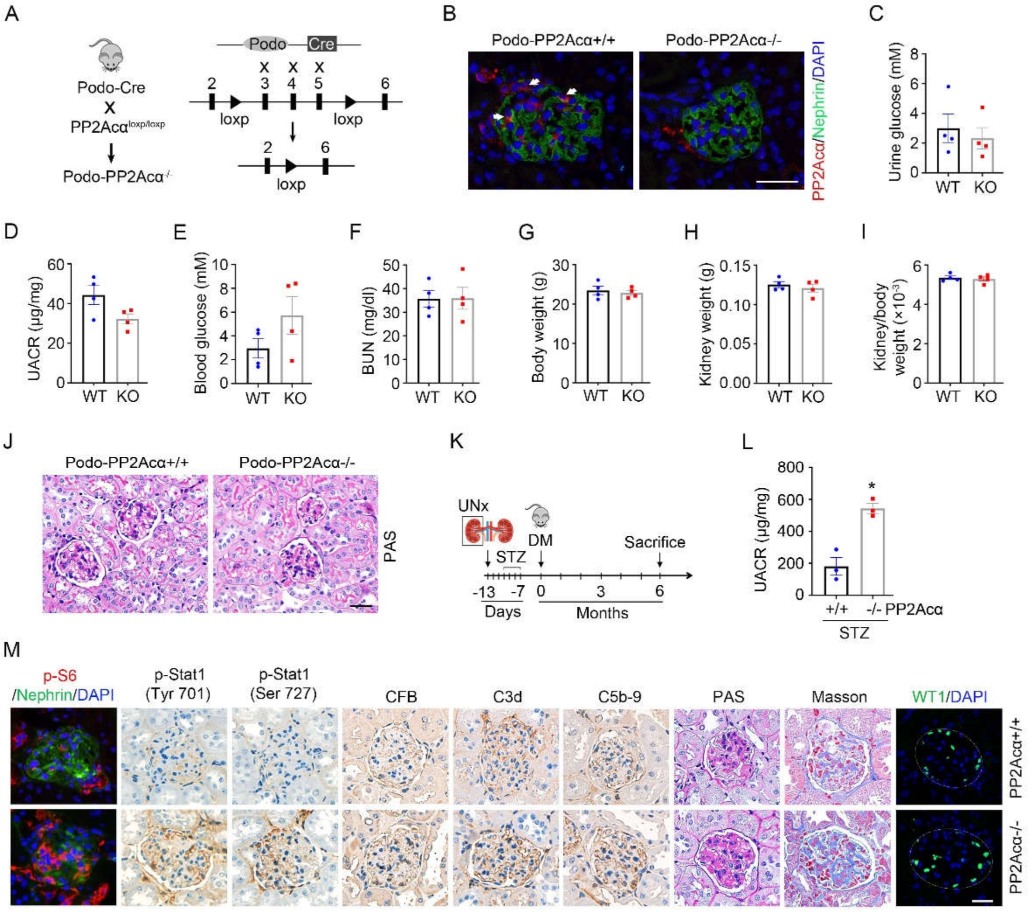

Figure S4. PP2Acα deficiency mediates high glucose-induced mTORC1 activation, CFB upregulation and DKD. (A) Schematic of the recombination strategy for generating mice with PP2Acα gene deletion in podocytes. (B) Immunofluorescence co-staining of anti-PP2Acα with nephrin. The arrows indicate the podocyte with PP2Acα staining positive in the kidneys. Scale bar =20 μm. (C-I) The graphs showing urinary glucose (C), UACR (D), blood glucose (E), the level of blood urea nitrogen (BUN) (F), body weight (G), kidney weight (H) and kidney/body weight ratio (I) between podo-PP2Acα$^{+/+}$ and podo-PP2Acα$^{-/-}$ mice at 2 months after birth. (J) Kidney histology as shown by periodic acid–Schiff (PAS) staining. Scale bar = 20 μm. (K) The strategy for STZ injection in podo-PP2Acα+/+ and podo-PP2Acα-/- mice. (L) Urinary albumin levels in podo-PP2Acα+/+ and podo-PP2Acα-/- mice 6 months after STZ-induced DM. *P<0.05 vs. podo-PP2Acα+/+ mice, n=3. (M) Representative immunofluorescent staining for p-S6, nephrin and WT1. Representative immunohistochemical staining images for p-Stat1 (Try 701), p-Stat1 (Ser 727), CFB, C3d and C5b-9 in glomerulus among different groups. Representative PAS staining and masson staining for diabetic kidney injury and kidney fibrosis among different groups. Scale bar = 20 μm. Data are expressed as the mean ± SEM. Comparison between the groups was performed using the two-tailed Student t test (paired t test).

Table S1. General diabetic patient information.

Supplementary Table 1: General information of diabetic patients.

NO.	Gender	Age	Diabetes type	HbA1c (%)	Scr (μM)	BUN (mM)	Urinary protein (g/24h)	UACR (mg/g)	eGF (m/min/1.73m^2)	Pathologic type
1	Female	59	II	8.1	122.9	10.6	5.556	2264	53.77	III
2	male	60	II	6.3	82.2	6.61	0.82	N/A	109.97	III
3	male	65	II	7.2	168.7	8.32	3.995	1314.6	37.97	IV
4	male	70	II	6.5	94.9	7.89	0.08	55.3	88.88	IIa
5	male	63	II	7.4	149.4	11.28	3	3441.9	46.82	IV
6	male	45	II	6.7	130.4	8.74	0.12	8.7	66.08	IIa
7	Female	52	II	8.4	81.1	13.41	14.531	3	95.56	IIb
8	male	58	II	4.8	222.7	22.43	4.98	2544.8	26.18	III
9	male	55	II	6.6	140.6	9.02	9.75	N/A	54.6	III
10	male	54	II	NA	169.8	15.42	1.173	59.9	40.53	IIa
11	male	61	II	5.4	115.8	6.57	5.159	N/A	71.47	III
12	male	52	II	8.2	96.3	8.14	5.068	N/A	98.76	III
13	Female	65	II	NA	157.5	8.9	17.241	14046.7	34.54	IIa
14	Female	47	II	NA	74.2	12.65	4.212	4743	103.22	III
15	male	48	II	NA	348.3	14.06	8.556	5891.3	16.1	III
16	Male	59	II	NA	139.1	8.65	0.84	N/A	54.06	IIb
17	male	63	II	6.5	107	6.79	0.176	43.9	79.36	I
18	male	66	II	7.1	116.1	7.31	5.71	6751.8	68.78	III
19	Female	39	II	7.8	128.8	6.02	7.01	N/A	57.22	IV
20	Female	57	II	NA	251.6	18.23	0.625	N/A	18.17	IIb
21	male	53	II	NA	169.9	13.95	0.138	9.8	40.77	IIa
22	male	52	II	6	135.8	9.71	5.831	2470.4	58.97	III
23	Female	45	II	9.7	76	3.86	1.801	2751.5	103.86	III
24	Male	65	II	5.7	108.5	3.65	3.575	3659.7	76.71	III
25	Male	38	II	NA	432.2	16.88	15	5539.7	13.96	III
26	Male	62	II	8.1	173.8	11.5	5.265	341.7	36.98	III
27	Male	50	II	8.6	126.9	6.7	0.868	318.7	66.71	IIa
28	Male	29	II	8.4	74.9	6.21	0.453	394.5	142.98	IIa
29	Male	49	II	11.2	97	5.32	24.683	10874.9	99.89	IIa
30	Male	41	II	8	463	18.95	5.273	3568.7	12.88	III
31	Male	45	I	6	191.5	9.31	9.283	9805.7	35.75	III
32	Male	47	II	6.8	270.9	10.97	8.112	4949.2	21.71	III
33	Female	65	II	8.8	82.6	5.38	0.254	161.3	86.25	IIb
34	Female	49	NA	NA	N/A	N/A	N/A	N/A	N/A	IV
Mean ±SD	Average	53.94 ±9.535		7.372 ±1.433	160.6 ±95.98	10.10 ±4.523	5.428 ±5.681	3308 ±3744	61.2 ±32.53	

Scr, serum creatinine; BUN, blood urine nitrogen; UACR, urine albumin to creatinine ratio ; eGFR, estimated glomerular filtration rate; N/A, not available.
